# Chronic traumatic encephalopathy: clinical‐biomarker correlations and current concepts in pathogenesis

**DOI:** 10.1186/1750-1326-9-37

**Published:** 2014-09-17

**Authors:** Sam Gandy, Milos D Ikonomovic, Effie Mitsis, Gregory Elder, Stephen T Ahlers, Jeffrey Barth, James R Stone, Steven T DeKosky

**Affiliations:** Departments of Neurology, Icahn School of Medicine at Mount Sinai, One Gustave L Levy Place, New York, NY 10029 USA; Departments of Psychiatry, Icahn School of Medicine at Mount Sinai, One Gustave L Levy Place, New York, NY 10029 USA; The Mount Sinai Alzheimer’s Disease Research Center, Icahn School of Medicine at Mount Sinai, One Gustave L Levy Place, New York, NY 10029 USA; James J Peters VA Medical Center, 130 West Kingsbridge Road, Bronx, NY 10468 USA; Geriatric Research Education and Clinical Center, VA Pittsburgh Healthcare System, Departments of Neurology and Psychiatry, University of Pittsburgh, Pittsburgh, PA 15213 USA; Naval Medical Research Center, Silver Spring, MD 20910 USA; Departments of Psychiatry and Neurobehavioral Sciences, University of Virginia, Charlottesville, VA 22908 USA; Departments of Radiology, University of Virginia, Charlottesville, VA 22908 USA; The Office of the Dean of the College of Medicine and Department of Nerurology, University of Virginia, Charlottesville, VA 22908 USA

## Abstract

**Background:**

Chronic traumatic encephalopathy (CTE) is a recently revived term used to describe a neurodegenerative process that occurs as a long term complication of repetitive mild traumatic brain injury (TBI). Corsellis provided one of the classic descriptions of CTE in boxers under the name “dementia pugilistica” (DP). Much recent attention has been drawn to the apparent association of CTE with contact sports (football, soccer, hockey) and with frequent battlefield exposure to blast waves generated by improvised explosive devices (IEDs). Recently, a promising serum biomarker has been identified by measurement of serum levels of the neuronal microtubule associated protein tau. New positron emission tomography (PET) ligands (e.g., [^18^ F] T807) that identify brain tauopathy have been successfully deployed for the *in vitro* and *in vivo* detection of presumptive tauopathy in the brains of subjects with clinically probable CTE.

**Methods:**

Major academic and lay publications on DP/CTE were reviewed beginning with the 1928 paper describing the initial use of the term CTE by Martland.

**Results:**

The major current concepts in the neurological, psychiatric, neuropsychological, neuroimaging, and body fluid biomarker science of DP/CTE have been summarized. Newer achievements, such as serum tau and [^18^ F] T807 tauopathy imaging, are also introduced and their significance has been explained.

**Conclusion:**

Recent advances in the science of DP/CTE hold promise for elucidating a long sought accurate determination of the true prevalence of CTE. This information holds potentially important public health implications for estimating the risk of contact sports in inflicting permanent and/or progressive brain damage on children, adolescents, and adults.

## Overview

**Figure 1:**
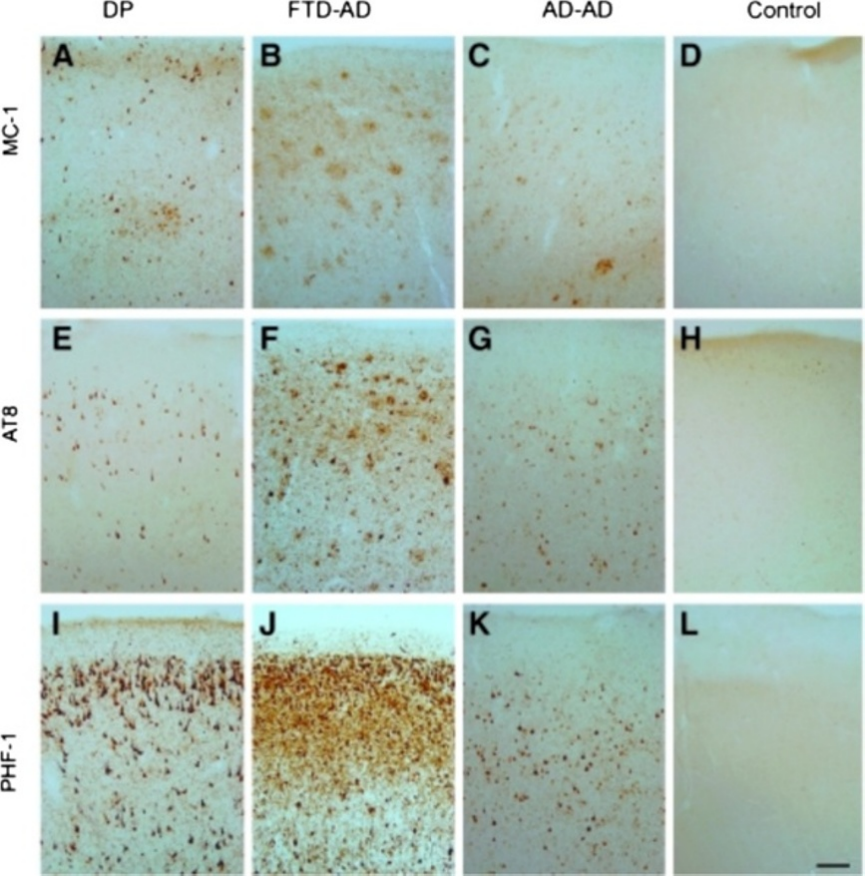
**Patterns of tau immunostaining in the frontal cortex of the patient with dementia pugilistica (DP), compared to Alzheimer’s disease (AD) and nondemented cases.** MC-1 immunolabeling of phosphorylated tau revealed neuronal labeling in the DP case **(A)**, plaque-associated neuritic labeling in the frontotemporal dementia (FTD)-AD case **(B)**, a mix of neurofibrillary tangles (NFTs) and dystrophic neurites in the typical AD case **(C)**, but absent in the control case **(D)**. AT8 immunoreactivity also was limited to intracellular NFTs in the DP case **(E)**, and within NFTs and plaque-associated dystrophic neurites in the FTD-AD case **(F)** and the typical AD case **(G)**, but were not present in the control case **(H)**. PHF-1 immunostaining was the most extensive of the three pathological tau markers, and showed significant intracellular and extracellular NFT labeling in the DP case **(I)**, and within NFTs and plaque‐associated dystrophic neurites in the FTD-AD case **(J)**, but was less extensive and limited to NFTs in the typical AD case **(K)**. No PHF‐1-positive tangles were observed in the control case (**L**; scale bar = 100 μm). From Saing *et al.*[5] with permission.

**Figure 2:**
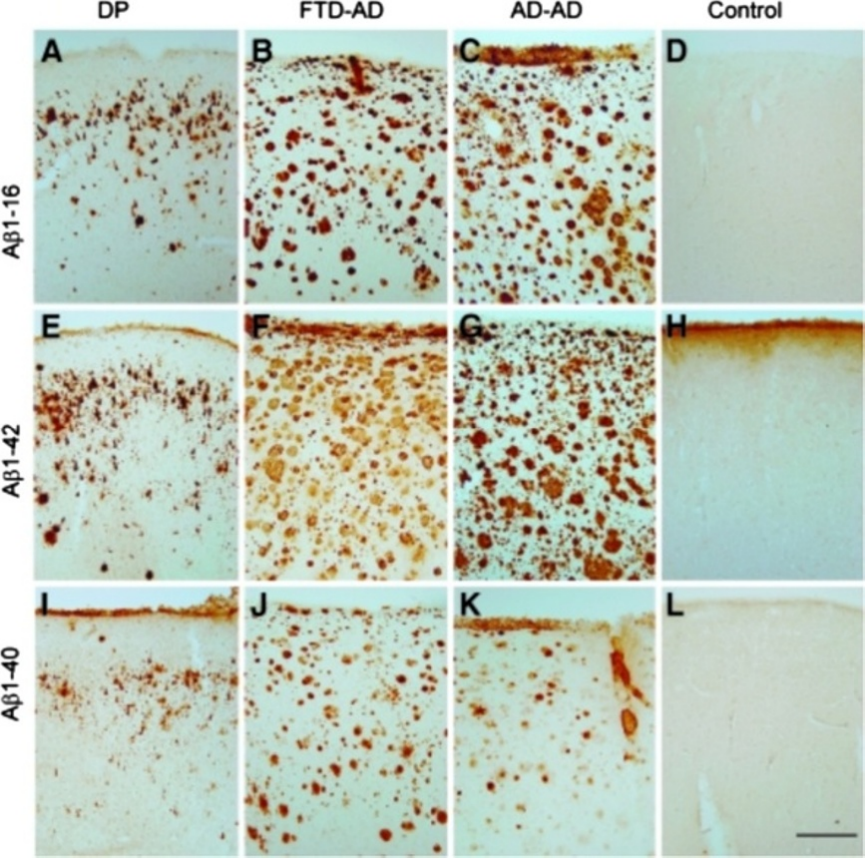
**Frontal cortical beta-amyloid (Aβ) neuropathology in dementia pugilistica (DP) as compared to that of Alzheimer’s disease (AD) and nondemented control cases.** Aβ1-16 immunostaining illustrates primarily diffuse plaque and extracellular neurofibrillary tangle (NFT) labeling in the DP case **(A)** as compared to the extensive plaque labeling seen in the frontotemporal dementia (FTD)-­AD case **(B)**, and the typical AD case **(C)**, but is absent in the control case **(D)**. Aβ1-42 immunostaining in the DP case **(E)**, the FTD-AD case **(F)**, the typical AD case **(G)**, and the control **(H)**, was similar to that observed with immunolabeling for Aβ1-16. Less Aβ1-40 immunolabeling was observed in the DP case **(I)**, with deposits being primarily seen within diffuse plaques and on extracellular NFTs. In comparison, Aβ1-40 was observed in plaques in the FTD-AD case **(J)**, and primarily within neuritic plaque cores and associated with blood vessels in the typical AD case **(K)**, and was absent in the control case (**L**; scale bar = 500 μm). From Saing *et al.*[5] with permission.

Chronic traumatic encephalopathy (CTE) is unique among brain diseases in having a history of decades of organized opposition to its codification as an authentic or valid entity. The conceptual entity has evolved over the 75 years since Harrison Stanford Martland [1], writing in *The Journal of the American Medical Association* in 1928, coined the term “punch drunk” to describe the tremors and impaired cognition that affected some boxers [1]. In 1937, Millspaugh [2] coined the term “dementia pugilistica” (DP), which was broadened to “chronic traumatic encephalopathy” (CTE) by Macdonald Critchley [3] in 1949. In 1973, John Corsellis and colleagues [4] brought DP/CTE into the modern day with their definitive documentation that progressive neurodegeneration (Figures 1 and 2) was associated with elective exposure to repetitive head trauma. This report set off a controversy that continues today regarding the role of society in regulating intentional head injury and in establishing the liability of organizations that encourage such exposure with little in the way of informed consent.

Despite condemnation of boxing by the American Medical Association [6] and by the American Pediatrics Association [7] in 1997 and predictions of the rapid demise of boxing, the sport continues today, and multimillion dollar purses still await the winners. In turn, the chance to compete for these purses continues to attract new boxers into professional associations, fueling the sport. Other sports associated with repetitive head trauma (e.g., ultimate fighting and mixed martial arts) have expanded and found larger participant groups and fans. Despite much social opposition to these sports, all efforts at boxing bans have so far been stymied by the influence of pro-boxing lobbyists on state and federal legislatures [8]. While over 50 sports-associated cases of CTE have been reported [9], efforts at educating the public regarding the risks of repetitive head injury are hindered by some retired boxing champions who refuse to recognize that their own brain disease is due to their chronic exposure to boxing. Newer “sports” involving various forms of fighting provide further encouragement.

**Figure 3:**
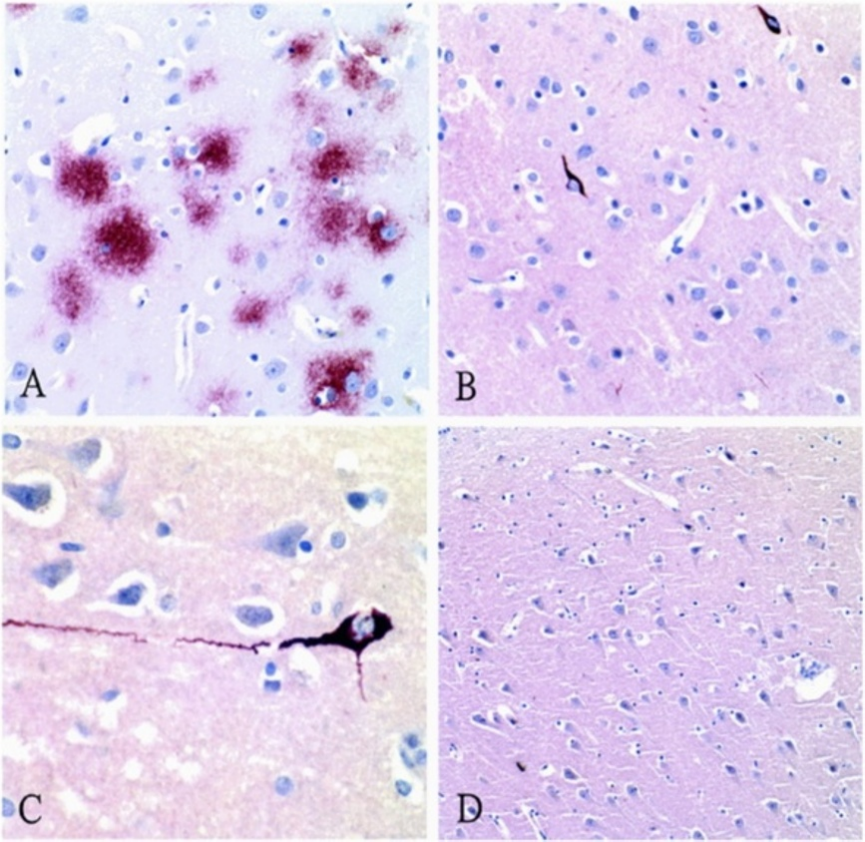
**Micrographs from the index case of CTE in an American football player.** Panel **A**, β‐amyloid immunostain of the neocortex (original magnification, ×200) showing frequent diffuse amyloid plaques. Panel **B**, tau immunostain of the neocortex (original magnification, ×200) showing sparse NFTs and many tau-positive neuritic threads. Panel **C**, tau immunostain (original magnification, ×400) showing an NFT in a neocortical neuron with extending tau-positive dendritic processes. Panel **D**, β-amyloid immunostain (original magnification, ×100) of the Sommer’s sector (CA-1 region of the hippocampus) showing no diffuse amyloid plaques. From Omalu *et al.*[10] with permission.

**Figure 4:**
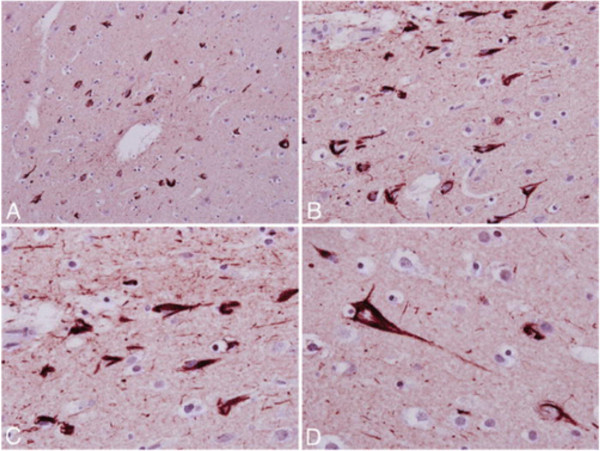
**The index case of military CTE.** Photomicrographs of tau-immunostained section of the frontal cortex showing frequent neurofibrillary tangles and neuritic threads **(A and** **B)**, with higher magnification **(C and** **D)** showing band- and flame-shaped neurofibrillary tangles and neuropil neuritic threads. Original magnification × 200 **(A)**, × 400 **(B)**, × 600 **(C and** **D)**. From Omalu et al. [15] with permission.

In 2005, Omalu and colleagues [10] revived the term CTE in their report of the index case of a retired National Football (NFL) player with progressive neurological dysfunction (Figure 3). The term CTE includes DP and supplants the use of the term DP. With the increasingly evident association of CTE with American football, the stakes grew exponentially. In part as a result of the NFL’s consistent denial of the danger of CTE to its players, in both public statements and legal challenge statements, the disease remained out of the spotlight until the League aligned itself with the independent and academic experts who were studying the disease [11]. CTE has also been associated with other high impact sports (soccer, hockey) and with exposure to improvised explosive devices (IEDs) in the battlefields of Iraq and Afghanistan [12–14] (Figure 4). Now, in the 21st century, the challenge is no longer the acceptance of the entity of CTE but rather a sorely needed accounting of the actual numbers of affected persons as well as the numbers of those who remain unaffected despite exposure to the identical repetitive head traumas. The role of aging has also gone unexplored. With those numbers in hand, we would be able to derive, for the first time, an accurate estimate of the true risk of each sport or type of military activity taken with consideration of the actual or estimated number of TBI episodes, without or with loss of consciousness. Accurate epidemiology, in turn, would facilitate the identification of risk factors for adverse cognitive outcomes, including the role of genetics in susceptibility.

**Figure 5:**
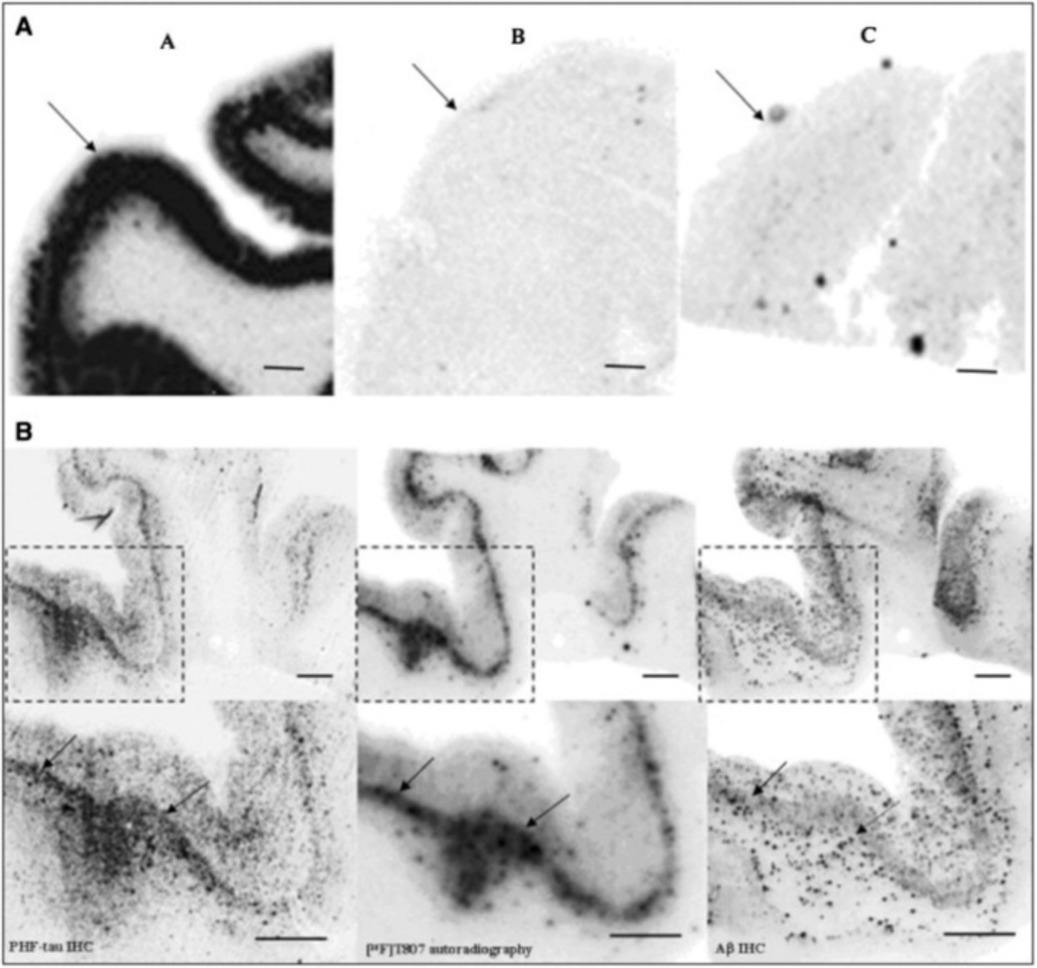
**[18 F] T807 autoradiography on brain sections and its comparison with paired helical filament (PHF)-tau and amyloid beta (Aβ) double immunohistochemistry (IHC). (A)** Representative images for [18 F] T807 autoradiographs from groups **A**, **B**, and **C** of brains ([18 F] T807 autoradiography, 20 μCi/section). Positive autoradiography signals were observed only in the gray matter of brain from the PHF tau rich group **A**. Arrows indicate gray matter. **(B)** [18 F] T807 colocalized with PHF-tau but not with Aβ plaques. (**B**, top row) Low magnification. (**B**, bottom row) High magnification from the framed areas. Images of PHF tau (left) and Aβ (right) IHC double immunostaining and autoradiogram image (middle) from two adjacent sections (10 μm) from a PHF-tau rich group **A** brain (frontal lobe). Positive [18 F] T807 labeling colocalized with immunostaining of PHF tau but not with Aβ plaques, as indicated by arrows. Fluorescent and autoradiographic images were obtained using a Fuji Film FLA-7000 imaging instrument. Scale bars = 2 mm. From Xia *et al.*[19] with permission.

Other key issues surrounding CTE diagnosis and research have emerged. While the boxers with DP were usually apathetic in disposition, those with sports and military TBI show prominent emotional dysregulation and sometimes violence, especially suicide [16]. This behavioral difference, while not yet completely analyzed, raises the question of whether abuse of steroids or other licit or illicit drugs might play roles in CTE [17]. This emotional dysregulation can include depression, anxiety, agitation, aggression, and a post-traumatic stress disorder-like (PTSD-like) clinical phenotype [18]. Since these are symptoms more likely to lead to psychiatric referral, a complete accounting of clinical CTE will require an alliance between neurologists (who frequently receive the dementia referrals especially in patients under 60 yrs of age), psychiatrists (who frequently receive the neuropsychiatric referrals), and neuropathologists (whose opinions are currently required to distinguish CTE from Alzheimer’s disease [AD] and other pathological entities). Neuroradiologists will play increasingly important roles as magnetic resonance imaging (MRI) methodologies and other neuroimaging biomarkers (i.e., amyloid imaging, tauopathy imaging; Figure 5) mature and are validated.

On the occasion of the first promising blood biomarker for TBI that correlates with outcome and the presentation of the first tauopathy PET images that support a diagnosis of CTE during life, we take this opportunity to review the existing knowledge about CTE up to now.

### Neuropsychology of CTE

The recommended assessment in CTE includes neuropsychological evaluation, neurological examination, brain imaging, and blood and CSF biomarkers. Particular attention should be paid to cognitive function, mood, personality, behavior, and olfaction [20]. Most of what is known about neuropsychological function in CTE comes from the study of boxers and, more recently, football players, athletes from other contact sports, as well as those exposed to domestic violence, and military personnel exposed to battlefield blast injuries [20, 21]. However, there have been no prospective studies linking clinical phenotypes during life (including neuropsychological function and outcome) with autopsy-confirmed CTE. Thus, the clinical and neuropsychological characterization of CTE is yet to be properly developed. The development of biomarkers for CTE (reviewed in detail below) should greatly facilitate the fulfillment of this goal.

In their review of 48 cases of neuropathologically confirmed CTE, McKee *et al.* [21] found that memory loss was reported in over half of the individuals. As in AD, loss of insight often precluded patients from recognizing their deficits, and this important piece of the presentation was derived from friends or family. Other studies have reported that impairment in executive function is common in neuropathologically confirmed CTE [22]. Executive functions are a collective set of higher order abilities (judgment, self-inhibitory behaviors, decision-making, planning and organization) considered to be primarily dependent upon adequate functioning of the frontal lobes of the brain. Damage to various regions of the frontal cortex can disrupt these higher order abilities, leading to poor impulse control, and socially inappropriate, avolitional, and apathetic behaviors. For example, damage to the orbitofrontal regions can result in significant changes in personality. Thus, changes in personality, apathy, impulsivity, aggression, and “short fuse” behaviors typical of CTE [23] are consistent with the atrophy and other neuropathological changes of the frontal lobes that have been described in nearly all reported cases of CTE [16, 21–24].

Neuropsychological, mood, and neurobehavioral dysfunction in CTE typically presents in midlife after a latency period, usually years or decades after exposure to the repetitive trauma. The cognitive and behavioral symptoms of CTE begin insidiously, followed by progressive and gradual deterioration. Mood symptoms are typically depression, apathy, irritability, and suicidality. Behavior symptoms are impulse control, disinhibition, and aggression as well as comorbid substance abuse [24]. The continued degeneration of brain regions most severely affected in CTE (cerebral cortices, hippocampi, amygdalae, basal forebrain, mammillary bodies) results in the worsening of behavioral and mood symptoms, and the further deterioration of cognitive abilities subserved by these regions. Baugh *et al.* [24] organized the neuropsychological and neuropsychiatric symptoms of CTE into the categories of cognition, mood, and behavior. As mentioned, the early cognitive symptoms of CTE involve memory impairment and executive dysfunction. The early involvement of hippocampal-entorhinal cortices and medial thalamic circuits may explain the memory impairment and its similarity to the memory loss associated with AD. As in AD, the genetic risk posed by apolipoprotein E ε4 (*APOE* ε4) may play a role in the dementia associated with CTE [25–27]. The dysexecutive syndrome in CTE may result from the early neurofibrillary degeneration of the frontal cortex [10, 21, 22]. As the disease progresses, there is worsening memory impairment and executive dysfunction, language problems, motor dysfunction, aggression (physical as well as verbal), and apathy [23]. Dementia is evident in most CTE individuals who live to be over the age of 65 years [24]. However, the high rates of suicides, accidents, and drug overdoses often lead to death prior to this age [10, 28]. As a result, most persons with neuropathologically confirmed CTE were not demented at the time of death. In view of the young age of onset as compared with AD, CTE may be misdiagnosed as the behavioral variant of FTD. However, CTE has a more gradual and prolonged progression than does FTD, and CTE is associated with a repetitive TBI history but with no family history [24].

Neuropsychological testing may be valuable in scientific studies on TBI in both the acute and chronic phases. Athletes and military combatants exposed to multiple blast events are at highest risk for sustaining multiple mild TBI or concussions and potentially developing CTE. In order to appreciate fully the effects of single and multiple concussive and sub-concussive brain injuries and to enable tracking of recovery in individual cases, brief baseline and serial post-concussion neurocognitive assessments, based upon the Sports as a Laboratory Assessment Model (SLAM) methodology, are the recommended standard of practice [29]. Key neurocognitive elements of these abbreviated assessments include attention, processing speed, reaction time, and learning and memory.

Once CTE is suspected, based upon a history of repeated trauma and presence of cognitive and/or behavioral impairment, scientific studies are needed to evaluate the value of comprehensive neuropsychological testing; e.g., assessment of general verbal and visuospatial problem solving, language fluency, attention, learning and memory, speed of mental processing, abstract reasoning, judgment, new problem solving, planning and organization, mental flexibility, sensory and motor intactness, and emotional/psychological and behavioral functioning. A recent cross-sectional study assessed cognitive impairment and depression, as well as the neuroimaging correlates of these dysfunctions, in former NFL players [30]. Former NFL players with cognitive impairment and depression were compared to cognitively normal retired players who were not depressed, and a group of matched, healthy control subjects. The cognitive deficits found were primarily in naming, word-finding, and memory (verbal and visual) and were associated with disrupted white matter integrity on DTI and changes in regional cerebral blood flow. Although none of the players fit the clinical profile of CTE per se, the sample size was very small. Nevertheless, this is one of the first studies examining the neural correlates of cognitive dysfunction and depression in players with a history of concussions (range 1–13 concussions in this sample).

### Neuropathological changes in boxers (dementia pugilistica, punch-drunk syndrome)

For this section, the combined term DP/CTE is employed, since the term DP appears in this original literature. However, the revival of the term CTE was intended to include DP and to supplant the use of the term DP. In 1934, Parker described three cases of boxers affected with the illness [31] and drew attention to the risk of developing DP/CTE in professional boxing. Neuropathological examination of these retired professional boxers’ brains demonstrated that the primary DP/CTE lesion involved multifocal intracellular aggregates of hyperphosphorylated tau, which resembled the neurofibrillary tangles (NFT) found in AD brains [32]. Using Congo red and silver staining, Corsellis and colleagues studied 15 cases of DP/CTE and confirmed the abundance of NFT. Like the NFT of AD, the NFT of DP/CTE is also reactive to antibodies to other AD-related proteins such as ubiquitin [33] and Aβ [34]. The anatomical patterns of NFT distribution are different in DP/CTE and AD: there is greater involvement of superficial layers of the associational neocortex in cases with DP/CTE and notably in the depths of cortical sulci [35]. This difference may reflect a unique way in which NFT are formed and propagated in DP/CTE.

In clear distinction to neuropathologically confirmed AD, no congophilic or argyrophilic plaques were observed in the Corsellis study [32]. In 1991, other investigators re-examined 14 out of the original 15 DP/CTE cases from the Corsellis study as well as additional brains of professional and amateur boxers, using Aβ immunohistochemistry and formic acid pre-treatment of tissue sections [34, 36–38]. Re-examination revealed that these 14 brains with NFT pathology (and even two amateur fighters’ brains that lacked NFT) also had Aβ-immunoreactive plaques [34, 36–38]. These DP/CTE plaques were described as diffuse in most cases; they lacked both Congo red positivity and the silver-positive dystrophic neurites that are typical of mature AD plaques. Some cases also displayed cerebrovascular deposits of Aβ. These data demonstrated that DP/CTE is associated with both the NFT and Aβ-related AD-like pathology. The antigenic similarities of neurofibrillary lesions in DP and AD led Roberts to suggest that the two conditions likely share the same pathogenesis and that TBI may be a risk factor for developing AD [37].

More recent studies reconfirmed that DP/CTE includes both NFT and Aβ pathology. Tokuda *et al.* [38] and Nowak *et al.* [39] examined the brains of boxers who suffered a progressive cognitive decline before death, and reported NFT as well as infrequent neuritic Aβ plaques. McKee and colleagues [21] studied two aged (80 and 73 years) professional boxers and presented them along with 37 previously published cases of boxers with neuropathologically verified DP/CTE. Similar to earlier reports, the most striking pathological feature in these two cases involved multifocal patches of neuronal and astrocytic NFT in superficial cortical layers, most frequently at the depths of the sulci and around large blood vessels [21]. In one of the two boxers, there were moderate diffuse Aβ plaques and sparse neuritic plaques in several neocortical regions. Collectively, these reports indicate that the neuropathology of DP/CTE was heterogeneous and involves both NFT and diffuse Aβ deposits, although the extent and proportions of these lesions vary from case to case and may be influenced by other factors such as number of years since TBI, age at the time of the TBI, and genetic predisposition. In a study by Geddes and colleagues, the brain of a 23-year-old boxer was found to have all neocortical areas affected with NFT; however no other changes (including Aβ deposits) were reported [40]. *APOE* ε*4* alleles have been associated with more severe cognitive deficits in boxers [25] and could contribute to more severe Aβ pathology, as has been demonstrated in AD. In an *APOE* ε*4* heterozygous retired boxer with DP/CTE, death was caused by hemorrhage due to amyloid angiopathy [41]. Clinical misdiagnosis of DP/CTE as AD is not unusual; interestingly, both boxers in the McKee report were diagnosed with AD during life. Differential diagnosis is likely to be clarified with amyloid imaging. We reported a case report illustrating this wherein the clinical diagnosis of retired NFL player could not be resolved until florbetapir imaging excluded the diagnosis of AD [42].

Other common findings in DP/CTE include cerebral infarcts and fenestrated cavum septum pellucidum (CSP) as well as substantia nigra degeneration [28]. Changes in substantia nigra could explain the increased incidence of Parkinsonism in retired boxers. It had been proposed that identification of CSP on CT scans could be of a diagnostic value for DP/CTE [43]. However this pathological feature is not consistently present [44], possibly because of the high prevalence of CSP in both boxers and non-boxers [45]. Because of the rigid enclosure of the brain inside the calvarium, acceleration-deceleration injuries associated with boxing often involve cerebral and meningeal vascular damage, the most common of which is subdural hematoma and to a lesser extent epidural, subarachnoid, and/or intraparenchymal hemorrhages [46]. Neuropathological sequelae of boxing appear to be less severe in amateur boxers, likely due to better-implemented regulations, fewer total bouts, and other measures of protection. In retired professional boxers, however, the risk of developing DP/CTE is greater: the extent of neurological abnormalities defined by CT and EEG, and severity of DP/CTE symptoms, correlated with numbers of fights and overall duration of boxers’ career [25, 47, 48].

Not shown here are some other CTE-associated structures described by McKee [21] including astrocytic tangles, perivascular tau pathology, or patchy tau pathology. An important significance for these structures is that McKee uses their presence to differentiate CTE from AD. The reader is referred to [21] for typical images of those structures.

### Neuropathological changes associated with American football

In contrast to the neurodegenerative changes associated with professional boxing, the chronic neuropathological sequelae of repetitive hits to the head in American football and other contact sports (hockey, rugby, etc.) have only been recognized more recently. Impacts to the head in American football cause less rotational acceleration than those suffered in boxing [49]; football-related injuries are of a translational acceleration-deceleration type, and despite the use of helmets, they can result in concussions or unconsciousness. In 2005, Omalu and colleagues reported the first neuropathology finding of CTE in a 50-year old retired NFL player [10]. In this case, they described AD-like changes that consisted of neocortical Aβ-immunoreactive plaques and sparse tau-immunoreactive neuronal and axonal aggregates resembling NFT and neuropil threads. While coexistence of Aβ plaques and NFT might have suggested an ongoing AD process, several observations argued against this idea: 1) there was no family history of AD; 2) at autopsy, the brain had no signs of cortical atrophy or overt neuronal loss; 3) the neocortical Aβ-immunoreactive plaques were numerous, but were diffuse (non-neuritic); and 4) NFT were scarce in the neocortex and absent in the entorhinal cortex and hippocampus which is the initial focus of NFT development in AD, prior to any cortical NFT [50]. The neuropathological pattern consisting of diffuse neocortical Aβ plaques and scarce NFT was similar to changes described in subjects with acute severe TBI [51] or in cases with preclinical AD, suggesting that both mild repetitive and severe brain injuries may initiate early AD-like changes. The second case reported by Omalu and colleagues [52] was a 45-year old retired NFL player who had numerous cortical and subcortical tau-immunoreactive NFT and neuropil threads, but no Aβ-immunoreactive plaques. In a more recent study, McKee and colleagues [21] described CTE in a 45-year old retired football player and compared their findings to previous neuropathology reports in four players who had played similar positions during their career. Tau-immunoreactive NFT-like neuronal and glial (fibrillar astrocytic) profiles and thread-like neuropil neurites were frequent in multiple cortical regions, predominantly occupying deep sulcal and perivascular locations [21]. NFT were also abundant in the entorhinal cortex, hippocampus, amygdala, nucleus basalis and septal nuclei, hypothalamus, thalamus, striatum, and olfactory bulb. Diffuse Aβ plaques were reported in 3 of the 5 cases [21]. As in CTE due to boxing (i.e., DP), there are similarities of the effects of football injuries to AD and other neurodegenerative disorders, notably involving NFT and diffuse Aβ deposition, and these findings require further investigation.

### Neuropathological changes associated with battlefield blast exposure

Studies of military servicemen who participated in recent wars indicate that repetitive mild head injury and concussions due to blast (explosive) injury might also result in CTE. This literature is the least mature since the index case of military CTE was only reported by Omalu a few years ago [15]. Brody and colleagues used diffusion tensor imaging (DTI) to evaluate the extent of axonal injury in 63 U.S. military personnel diagnosed with mild TBI after blast exposure with secondary mechanical injuries [53]. They found that, in these subjects, TBI was associated with significant axonal injury, which was still present 6–12 months later at follow up evaluation with DTI. Diffuse loss of white matter integrity was also detected using high angular resolution diffusion imaging (HARDI) in military veterans from Iraq and Afghanistan who had been diagnosed with mild TBI and comorbid PTSD [54]. Collectively, these findings indicate that TBI associated with blast or chronic mild injury produces chronic neuronal damage. Blast injuries and closed head injuries have similar clinical features [55], however, the extent of neurological damage in blast injured veterans is likely influenced by injuries of other systems, since air-filled organs (including lungs) are often severely affected [56, 57].

Goldstein [58] and colleagues performed postmortem analyses of brain tissues from four military veterans who were exposed to blast or concussive injury and compared them to four professional athletes who suffered repetitive concussions. In the neocortex from military veterans, there were frequent perivascular and deep sulcal accumulations of NFT-like tau-positive neurons and glial cells as well as dystrophic axons were reported [58]. Subcortical white matter in close proximity to these lesions had dystrophic axonal changes and clusters of activated microglia were described. These pathological changes were similar to those observed in sports CTE. The findings in this study have been challenged because all military CTE subjects had histories of *both* civilian and military TBI, making it impossible to distinguish which lesions arose from which injuries. Similar tau-immunoreactive neuropathological features were described in an Iraqi war veteran with PTSD who had been exposed to multiple blast explosions during his deployments [15]. These autopsy studies of brains from blast-injured veterans are not in agreement with recent body fluid biomarker analyses in soldiers exposed to blast [53], emphasizing both the variability of such injuries and the variability in the accuracy of the historical details surrounding the injuries. In this study of Army officers exposed to different levels of blast overpressure by firing heavy weapons, Blennow and colleagues [59] measured CSF biomarkers of neuronal injury (tau and neurofilament protein) or glial injury (GFAP and S-100β) as well as CSF/serum albumin ratio, hemoglobin, and bilirubin content in CSF. GFAP and S-100β were also analyzed in serum samples. All analyses indicated normal levels of examined markers, leading the authors to conclude that high-impact blast was not associated with biomarker evidence of brain damage [59]. The discrepancy between this report and published evidence of neuropathology in blast injured servicemen warrants further investigation, and the issue of whether the blast wave itself causes injury remains open. Another possible explanation is the lag time between injury and serum sampling. Both [53] and [59] illustrate the discrepancy between neuropathology that can be static and long-lasting vs serum biomarkers that will rise acutely and then fall as the marker molecule is cleared. Recent studies of serum tau as a marker of acute TBI in hockey players showed surprisingly good correlation with outcome [60].

### Molecular pathogenesis of CTE

As noted above, CTE is considered to be primarily a tauopathy with frequent coexistence of Aβ pathology. Notably acute severe TBI is associated with diffuse Aβ plaques and minimal tau pathology [51]. Other types of abnormal protein aggregates are less common in CTE; for example, alpha-synuclein immunoreactive inclusions were not reported in any of 51 CTE cases reported by McKee and colleagues [21]; however, they were detected only following acute severe TBI [51]. Multiple cases of Parkinsonism have been reported in professional boxers, and there is increased prevalence of ALS in professional football [61] and soccer players [62].

The detailed molecular pathogenesis of CTE is unknown. As observed in cases with single severe head trauma, axonal injury occurs in CTE, and this may be responsible for accumulation of phosphorylated tau. Tau is a microtubule-associate protein which can undergo excessive phosphorylation in the pathological milieu of brain injury associated with ischemic foci that increase the risk of damage from oxidative stress. The idea of ischemic changes as a contributing factor to tau hyperphosphorylation and formation of NFT-like changes in CTE is consistent with their preferential deep sulcal and perivascular localization. Because both neuronal and astrocytic NFT are observed in these areas, it is possible that changes in tau are related to a more general injury response mechanism that is not injury-specific or cell-specific (e.g., brain ischemia, inflammatory reaction).

Diffuse axonal injury (DAI) is the most consistently reported neuropathological feature following acute head trauma [63]. DAI is associated with intra-axonal accumulation of tau, Aβ-precursor protein (APP), ubiquitin, and α-synuclein [64, 65]. As an acute reaction to TBI, there is an upregulation of APP and/or a switch in its metabolic processing resulting in increased concentrations of Aβ, which can deposit in diffuse, predominantly Aβ42, plaques both acutely after severe TBI [51] and chronically in boxers with DP/CTE. In studies of both autopsy cases and in surgical biopsy tissue from subjects with TBI [51], about 30% of acute severe TBI patients are reported to show evidence of Aβ plaques. Aβ deposits in CTE have received less attention in the literature than has the tauopathy; however these changes may have a significant role. The development of Aβ lesions may depend on age of the subject, time interval since the head injury, and genetic factors. More extensive and mature Aβ pathology (i.e., neuritic plaques) may be absent, or may recede over years, in subjects with protective genetic factors that can result in better degradation/clearance of Aβ from the brain [66, 67], while tau-related pathology may develop more slowly, may be more resistant to degradation and therefore observed at late stages of disease. Other genetic factors (*APOE4*) may influence Aβ pathology. The link of *APOE ε4* to dementia in CTE is unexpected on the basis of tauopathy, since *APOE ε4* modulates Aβ pathology in AD but not tauopathy. Perhaps *APOE ε4* modulates the severity of the acute Aβ deposition which, in turn, affects clinical severity. This is a key point in determining which types of interventions will be most promising and at what point in pathogenesis each is most likely to be successful. Recent evidence that chronic action of interleukin-1β can reduce amyloidosis while exacerbating tauopathy raises the possibility that this neurodegeneration-associated cytokine may drive the conversion of acute post-traumatic Aβ deposition to chronic tauopathy with only minimal or inconsistent Aβ residua [68].

Alternatively, the extent and type of neuropathology changes in CTE resulting from different contact sports may reflect differences in the biomechanics associated with head injury, including impact level (force) and type of head movement (rotation, acceleration, deceleration). Novel genetic, biomarker, and neuroimaging analyses should be developed and employed for early detection of neuropathology, to provide a window of opportunity for preventing or at least delaying the clinical manifestations of neurodegenerative disease after TBI. Furthermore, timely identification of people at risk (e.g., *APOE ε4* carriers) [27], and regular testing (imaging, CSF analyses, and detailed neuropsychological evaluation) should be considered essential.

### Modeling CTE in laboratory animals

**Figure 6:**
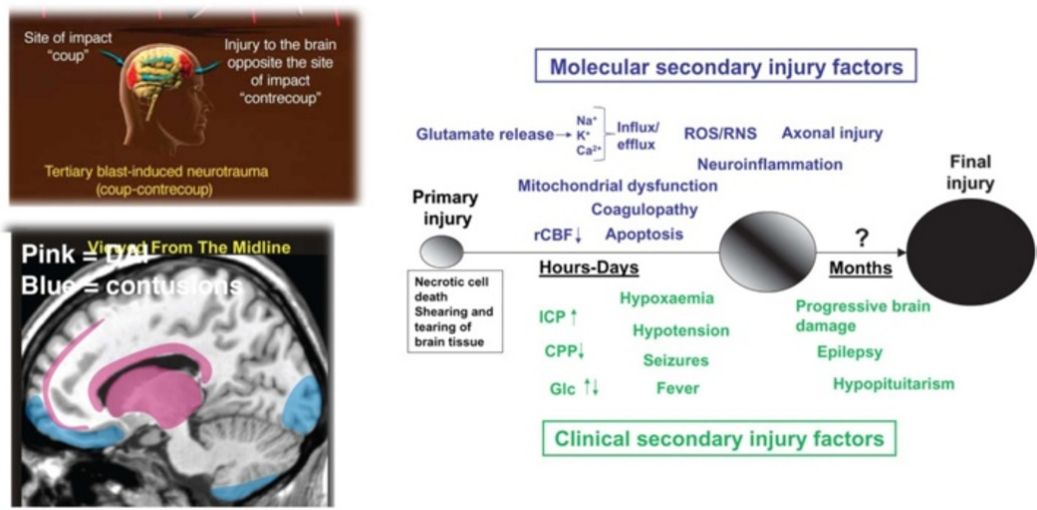
**Molecular pathogenesis of TBI and CTE.** The upper left panel shows the typical sites of coup/contrecoup injury as occur in blast as well as other types of closed head injuries. The lower left panel illustrates the most common locations for diffuse axonal injury (pink) and contusions (blue) following closed head injuries. Reproduced with permission from Taber *et al.*[80]. The large panel on the right illustrates current concepts of mechanisms underlying primary and secondary injury mechanisms in TBI. At early times after injury, glutamate release and ionic disturbances (Na+, Ca2+ and K+) disrupt energy metabolism and cause other metabolic disturbances that lead to decreases in cerebral blood flow. Mitochondrial dysfunction causes increases in reactive oxygen (ROS) and nitrogen species (RNS) that can cause further cellular injury. Tissue damage evokes neuro-inflammatory changes that emerge later. Injury may be exacerbated by secondary clinical factors including hypoxemia, hypotension, fever and seizures. These secondary molecular and clinical factors lead to progressive tissue damage. Abbreviations: Ca2+, calcium ions; CPP, cerebral perfusion pressure; Glc, Glucose; ICP, intracranial pressure; K+, potassium; Na+, sodium; rCBF, regional cerebral blood flow. Reproduced from Marklund *et al.*[70] with permission.

Currently there is no experimental animal model that recapitulates all pathophysiological aspects of human CTE. The best understood pathophysiological mechanisms associated acutely with severe blunt impact TBI are hemorrhage, mechanical tissue damage, and diffuse axonal injury (DAI) [69]. DAI results when angular forces cause shearing or stretching of axons; this can impair axonal transport which is manifested pathologically by focal axonal swellings, most commonly at gray/white matter junctions particularly in frontal and temporal cortical regions. Contusions occur as the result of coup/contrecoup injuries most commonly affecting the frontotemporal and occipital cortex. However not all injury occurs at the time of initial impact. Rather a complex cascade of pathophysiological effects unfolds over the ensuing hours and days following injury and results in further tissue damage [70] (Figure 6). Factors thought important in this secondary injury cascade include release of excitatory amino acids such as glutamate, calcium dyshomeostasis, mitochondrial dysfunction and oxidative stress. Increased glucose utilization at a time of decreased cerebral blood flow also likely exacerbates injury. While these molecular mechanisms are thought to be operative in moderate to severe TBI, there is much less in the way of neuropathological or molecular data on mild TBI and particularly from the mild repetitive concussions that contribute to CTE. What sets CTE apart from other injuries is a relentlessly progressive course leading to a syndrome that continues to progress even in the absence of further head trauma. Thorough understanding of this pathological transition in CTE would be facilitated greatly by an appropriate animal model.

### Relevance of existing animal models of TBI to human CTE

Because of its potential clinical importance, animal modeling of TBI has been vigorously pursued and a number of methods have been developed that can induce focal, diffuse or mixed brain injury [70]. Most studies of TBI use rodents, but rabbits, pigs, cats, dogs, and nonhuman primates have all been studied as well [69]. The choice of species has practical as well as theoretical implications. While rodents are less expensive than larger animals, their lissencephalic brains lack the structure of gyri and sulci found in humans. The white/grey matter ratio is also less in rodents compared to human brain; these anatomical factors may affect the biophysical properties of the brain’s reaction to mechanical injury. Species such as pigs and non-human primates offer the advantage of having a brain more similar to humans, but their cost and in the case of non-human primates availability limit their wider use for TBI research.

To model various types of TBI in experimental animals, several methods have been developed [70]. Among the more widely used models, controlled cortical impact (CCI) produces focal contusions with pericontusional axonal injury while fluid percussion models produce more diffuse axonal injury. Weight drop methods can produce a range of injuries depending on the force applied (mass x distance of the drop), and whether an open skull or closed skull technique is used. There has been recently increased interest in models of blast injury, due primarily to its importance in military head trauma [71]. Importantly, these models are associated with significant deficits in cognitive and motor function. In addition, anxiety and depression are common features of the postconcussion syndrome and evidence for impairment in both domains have been found as sequelae of TBI in some animal models [72]. There are several recognized limitations of the animal models. While human TBI represents a heterogeneous injury, most animal models try to replicate more isolated pathological factors [69]. Human TBI is frequently accompanied by hypoxia, hypotension and ischemia, factors that are not prominent in many animal models [69]. Accordingly, TBI models can be utilized in combination with additional insults such as hemorrhagic shock which often accompanies blast TBI [71]. Another limitation of traditional TBI models in replicating CTE is that most produce relatively severe focal or diffuse damage that is more similar to the type of injury found in moderate to severe rather than mild TBI. Significant efforts have been made to adapt these models to produce effects of mild TBI. For example, the CCI device has been modified to deliver impacts to the intact mouse skull resulting in a more mild TBI-like outcome [73]. The weight drop technique has also been used to produce repetitive mild TBI in the mouse [74, 75], and the lateral fluid percussion model has been modified to deliver milder injuries in the rat [76]. Additional models have been developed to mimic mild TBI in combat conditions including a closed‐head projectile concussive impact in rats [77], and a model of blast induced repetitive mild TBI in the rat that has been found to induce chronic behavioral changes [78, 79].

Animal models of single‐episode TBI have been valuable in determining the time course and the extent of cytoskeletal derangements which are a key feature of CTE. In both humans and experimental animals, TBI-induced axonal damage is associated with extensive accumulation of multiple proteins [65, 81]. Axonal microtubule and neurofilament (NF) proteins are altered acutely after TBI [82], and they are more pronounced following moderate injury [83]. In pigs subjected to a rotational acceleration injury, diffuse axonal damage is characterized by co-accumulation of tau and Aβ together with NF and APP throughout the white matter, while in cortical regions phospho-tau aggregates appear in axonal bulbs as well as in structures resembling NFT [81]. Similar changes occur in rodent models of TBI in which alterations in tau and other microtubule associated proteins can be rapid and transient [84, 85]. For example, in rats subjected to a mechanical compression injury increased tau phosphorylation occurs in cortical regions in as little as 10 min following injury but then is undetectable by 12 hours [84]. Transient elevations of serum and CSF tau can also be detected following TBI in rats [86, 87]. More sustained increases in a cleaved form of tau have been found in the cortex and hippocampus of rats following a CCI injury [85], and increased phospho-tau has been observed in cortex of mice 30 days after 5 mild TBI injuries were delivered utilizing a weight drop device [75]. A single blast-induced TBI resulted in acute (up to two weeks) axonal injury in the form of silver impregnated neuronal processes in rats [88] and increased levels of phosphorylated tau were detected in NFT like lesions in mice [58]. In rats, increased levels of tau have been found in multiple brain regions at 71 days following a blast injury [89]. Thus acute to subacute changes in tau are common across a spectrum of experimental TBI models.

However, few studies have yet revealed whether a subsequent chronic neurodegenerative changes are induced. Indeed the only study to our knowledge that has addressed this issue is the study by Hoshino *et al.* [90] who studied the chronic effects of fluid percussion injury in rats. They reported a progressive loss of cortical neurons over a six-month period accompanied by appearance of increasing numbers of phospho-tau immunoreactive neurons [90]. As noted above, rodent models of TBI are widely used because of their accessibility and relatively low cost. However, in addition to the anatomic differences between rodent and human brain, rodent models for CTE are potentially limited by the fact that mice and rats do not readily develop human like neurofibrillary pathology, a key histopathological feature of CTE. Rodents and humans differ in life spans, and species differences in tau also contribute to the difficulty of inducing NFT like lesions in rodents. The lack of human-like NFT pathology in rats and wild type mice has in particular been a limiting factor in the development of CTE models and has led to attempts to create more human like tauopathy models by introducing human tau transgenes into mice.

The non-traumatic neurodegenerative diseases that most closely resemble CTE pathologically are some forms of frontotemporal dementia (FTD). While most FTD cases can occur without evidence of family history, in some families the disease is inherited and caused by mutations in the tau gene on chromosome 17 [91]. Mice expressing either human wild type or FTD-‐mutant tau develop a neurodegenerative disease spontaneously and accumulate NFT‐like lesions with aging [92]. These lines have been used to investigate tauopathy‐related dementias and offer potentially relevant models for CTE [92]. However to date only limited use has been made of these mice to study TBI. One study [93] examined the effects of mild repetitive TBI using a CCI injury in transgenic mice expressing the shortest human tau isoform. Transgenic and non-transgenic mice were subjected to a total of 16 mild TBI injuries over a period of 4 weeks and animals were examined histopathologically at 9 months after injury. Despite this aggressive injury protocol there was no general effect on histopathology or behavior of the tau transgene other than in one of the 18 transgenic mice that showed behavioral deficits and developed extensive telencephalic NFT like lesions and cerebral atrophy. In contrast, increases in total and phospho-tau immunoreactivity have been seen following a more severe CCI injury in JPNL mice which express the four repeat form of human tau containing the P301L mutation associated with FTD [94]. In another study, mice that expressed the 6 isoforms of human tau and lacked murine tau were subjected to a single or repetitive mild TBI using an electromagnetically controlled impactor; three weeks later, phospho‐tau immunoreactivity, neuronal injury, and glial reaction were more pronounced in the group with repetitive mild TBI [95].

As discussed above, CTE is a heterogeneous disease with inconsistent pathology including not only tau but also Aβ lesions. Accordingly, several studies have investigated the effects of TBI in 3xTg-AD mice [94, 96, 97]. These mice also express the P301L mutation in tau associated with FTD but in combination with APP and presenilin mutations associated with familial Alzheimer’s disease. With aging these mice develop increased Aβ40 and Aβ42 levels, accumulate intraneuronal Aβ and exhibit amyloid plaques and NFT like lesions, thus exhibiting a more complete spectrum of AD like pathology when compared to other transgenic mouse models. Controlled cortical impact injury in 3xTg-AD mice accelerated the development of tau abnormalities [96] with increased phospho‐tau immunoreactivity seen histologically in the days following injury [96]. Tau changes persisted for at least 7 days after injury and were associated with intra-axonal accumulation of several kinases that phosphorylate tau [96]. In agreement with previous reports in other transgenic mouse models [98–100], the CCI injury in 3xTg-AD mice also resulted in intra-axonal Aβ accumulation [96]. However, in this model combining tauopathy and amyloidosis, the anatomic pattern and time course of changes in Aβ and tau were distinct [96], and treatment with a γ‐secretase inhibitor blocked posttraumatic Aβ accumulation but had no effect on tau pathology suggesting that once initiated these two pathologies may take independent courses of progression [96]. In contrast, reports from Loane *et al.* indicate that Aβ-reducing agents prevented TBI-induced neurodegeneration [101, 102].

Better animal models of CTE are an urgent need given that to date no injury in animals has replicated the full spectrum of pathological findings in human CTE. Acute to subacute changes in tau are common across a range of experimental TBI models suggesting that injured animals can manifest at least some initial cytoskeletal changes observed in brains of humans with CTE. However most studies have focused on acute effects of TBI and greater attention should be directed to determining whether a chronic neurodegenerative condition can be induced after TBI in animals. As mild TBI and concussions draw increasing attention as the most common type of civilian and military TBI, future studies of repetitive concussions in both rodent and non-rodent species will be needed to determine the optimal model.

### Neuroimaging of CTE

Identification of specific imaging biomarkers for CTE has been challenged by the lack of correlation between any available neuroimaging studies and post-mortem tissue assessments demonstrating histopathologic hallmarks of CTE. To date, no systemic *in vivo* biological markers have been identified that reveal the presence of CTE. Given that most case reports of CTE involve individuals with a history of repetitive sports-related trauma, the neuroimaging portion of this review will focus upon abnormalities present in athletes with a history of concussion.

Common structural abnormalities often reported in the brain of professional boxers include cavum septum pellucidum (often with fenestrations), ventricular and sulcal enlargement, and cortical and cerebellar atrophy. Although CSP is often reported in studies of boxers and is postulated to be a sign of DP/CTE [43], and a marker of brain atrophy [44, 103, 104], its relatively high incidence in the normal adult population precludes its presence as a diagnostic specific to boxing [105]. Despite the lack of specific *in vivo* findings in CTE, studies using both traditional and newer imaging methods have advanced our understanding of the consequences of repetitive head trauma. Newer imaging modalities and the use or development of advanced analytical methods (e.g., ref [106]) have revealed brain abnormalities in repetitive head trauma where more traditional methods found none, suggesting that comprehensive evaluation of CTE should include neuroimaging as part of an overall clinical assessment.

#### Structural Imaging

The major traditional structural imaging techniques used to evaluate brain function in boxers, and more recently in football players, are computed tomography (CT) and magnetic resonance imaging (MRI). In acute settings, these standard, structural imaging techniques have clinical utility in ruling out other acquired brain disorders such as tumor or stroke and have been utilized to rule out potentially fatal consequences of acute TBI such as hemorrhagic contusion, subdural and epidural hematomas, and subarachnoid hemorrhage. However, structural (or functional for that matter) imaging findings alone are not sufficient to diagnose CTE or determine who might eventually develop CTE. In addition, some of the structural imaging changes identified in CTE are similar to those seen in AD [107], and there may be similarities as well with normal aging. Early studies demonstrated that CT might not be as effective as MRI in identifying injury in the acute phase [108]. In non-acute settings, high-field MRI may be the preferred modality in the evaluation of TBI due to its improved tissue clarity and contrast of pathology, and because it eliminates the risk of ionizing radiation associated with CT [103, 107, 109]. These features of MRI make it a valuable diagnostic tool for determining the extent of chronic TBI [107].

In view of the improved tissue contrast and additional biologically relevant information afforded by MRI, early studies began to investigate whether MRI was superior to CT as an imaging modality in boxers. A study conducted in 1990 did not demonstrate MRI superiority to CT in 47 retired amateur boxers as compared to two age-matched control groups: soccer and track and field athletes [110]. However, another study published that same year found that some abnormalities detected by MRI were not shown by CT [103]. There were several limitations in these early studies, including comparison of professional boxers to amateur boxers, who may not show neurologic damage because of their limited number of exposures, and incomplete information in professional boxers in terms of number of years spent boxing or the number of bouts fought. In addition, most studies were cross-sectional, thus it cannot be determined from these studies whether the abnormalities were present before or the result of, boxing [105]. It should be noted that both MRI and CT have seen dramatic technological improvements over the past two decades.

**Figure 7:**
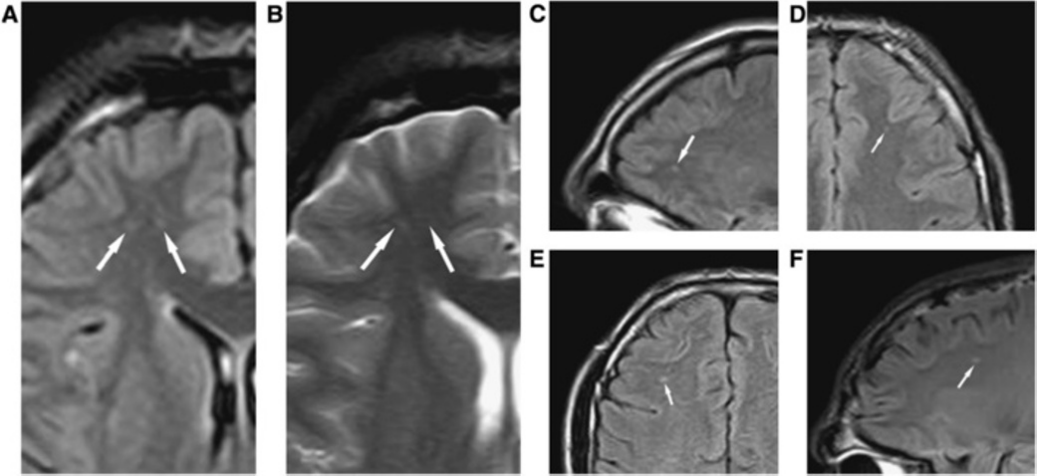
**Diffuse axonal injury on MRI.** (Panels **A–F**) Typical diffuse axonal injury, indicated by arrow. DAI was defined as focal areas of abnormal increased signal intensity on FLAIR and T2-­weighted sequences, measuring up to 5 mm in maximum diameter, and located at the gray matter = white matter interface or within or adjacent to the corpus callosum. In the Orrison *et al.* sample, 29% had DAI. Reproduced Ïrom Orrison *et al.*[107] with permission.

**Figure 8:**
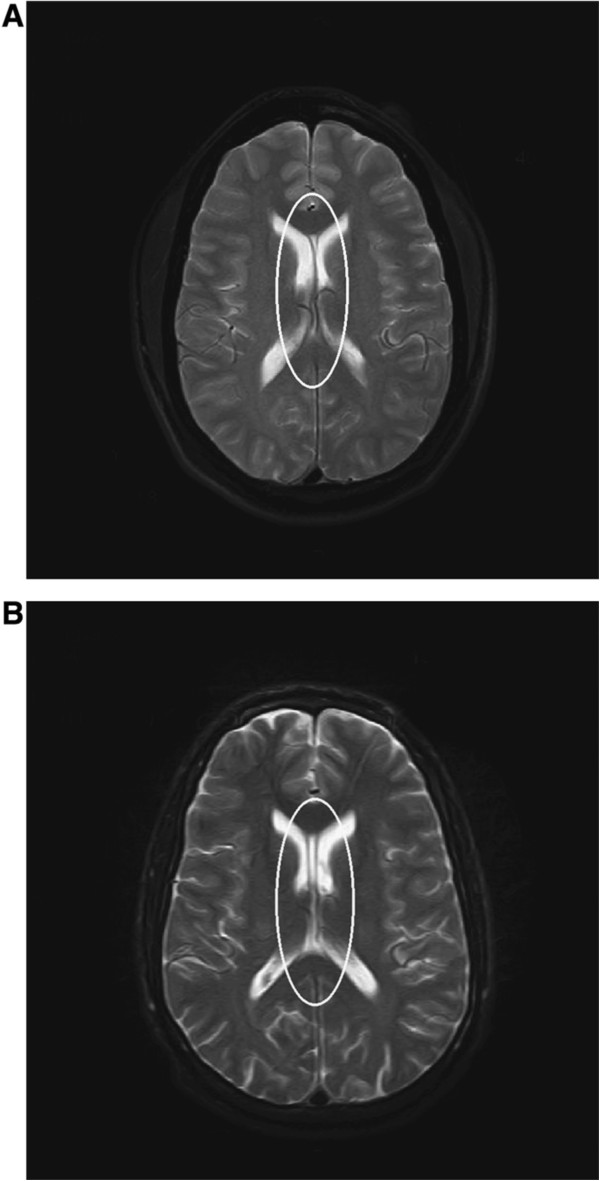
**Cavum septum pellucidum (CSP) on MRI. (A)** Mild **(B)** Moderate. No severe CSP was observed. Orrison et al. [107] examined one hundred consecutive unselected MRI scans that were performed on professional unarmed combatants (boxers and mixed martial arts fighters) in two outpatient imaging settings. Seventy-five were imaged on a 1.5-Tesla (T) MRI system and 25 were imaged on a 3.0-T high field MRI system. CSP was defined as the presence of a fluid filled space separating laminae of the septum pellucidum. CSP was graded as mild, moderate, or severe. CSP was found in 42% of subjects, due in part due to the improved resolution of the higher field strength MRI systems used.

A recent review of the MRI literature [107] used a systematic checklist approach to assess 100 unselected consecutive 1.5 and 3.0 Tesla MRI examinations of professional boxers to determine the extent of identifiable TBI findings. Results indicated that 76% of boxers had at least one finding associated with TBI: 59% had hippocampal atrophy, 43% CSP, 32% dilated perivascular spaces, 29% diffuse axonal injury, 24% cerebral atrophy, 19% increased lateral ventricular size, 14% pituitary gland atrophy, 5% arachnoid cysts, and 2% had contusions [107] (Figures 7 and 8). Statistical relationships were found between number of bouts and lateral ventricular size, and years of fighting correlated with dilated perivascular spaces and diffuse axonal injury. An earlier systematic review of the literature from 1985 to 2000 [111] on concussion in a variety of contact sports, found that male boxers had the highest frequency of concussion at the recreational level [111]. While these reviews indicate significant brain abnormalities in boxers exposed to frequent concussive and subconcussive blows to the head, prospective studies will be needed to determine whether the underlying process is degenerative and progressive in nature and related to and consistent with CTE clinical outcomes.

#### Diffusion tensor imaging

**Figure 9:**
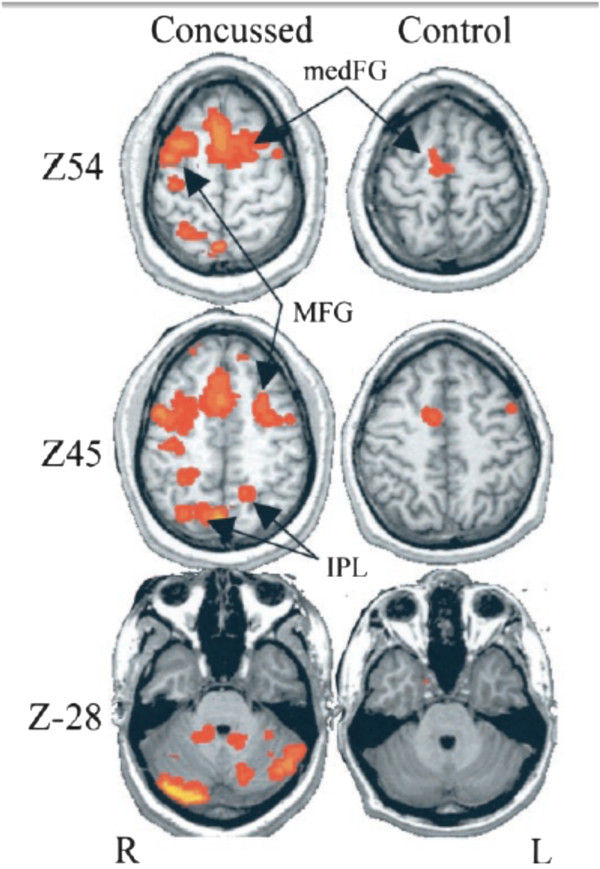
**BOLD imaging was acquired from college football players while performing a bimanual sequencing task.** The “concussed” group consisted of four individuals who were within one week of sustaining a concussion. The “control” group included the additional four players who did not receive a concussion, with imaging acquired post‐season. Regions of significantly increased activity during performing of the bimanual sequencing task are seen within the brains of those individuals who sustained a concussion as compared with controls. Images and data are reproduced from Jantzen *et al.*[112] with permission.

Newer imaging techniques such as diffusion tensor imaging (DTI) (Figure 9) are more sensitive for identifying the presence of axonal injury that occur due to the shearing forces of TBI. DTI is a relatively new technique and provides information reflecting the integrity of white matter fiber tracts.

Quantitative values used for spatially mapping DTI include apparent diffusion coefficient (typically mean diffusivity, MD), which measures the magnitude of the diffusion of water molecules, and fractional anisotropy (FA) or relative anisotropy (RA), which measure the directional preference of water molecules throughout brain fibers. White matter tracts normally constrain the isotropic diffusion of water. An FA value approaching 1.0 reflects maximal anisotropic diffusion, and values approaching zero indicate compromised white matter integrity. In addition to FA and RA, axial diffusivity and radial diffusivity describing magnitude of diffusion longitudinal and transverse to the dominant direction of white matter tracts may also be derived from the calculated diffusion tensor. DTI values are affected when there are changes to the microstructure of brain tissue. Disruption of axonal integrity due to shearing forces associated with head trauma results in a general reduction of FA values as the normal barrier to isotropic diffusion is altered. This type of information is not available from routine MRI; thus, white matter abnormalities that may be present due to repetitive TBI often go undetected with routine imaging.

Chappell *et al.* [113] used a voxel-based analysis of DTI data to examine the ADC and FA in professional boxers and found previously unreported abnormalities that were assumed to reflect cumulative brain injury resulting from non-severe, repetitive head trauma [113]. Regions were found to have increased ADC, decreased FA, and decreased ADC in gray matter in the boxer group compared to a group of controls with no neurological history. The regions affected included lower splenium and cortical regions laterally and dorsolaterally in the frontal and posterior lobes. The authors concluded that their findings represented evidence that sustained boxing activity causes structural abnormalities in the brain.

**Figure 10:**
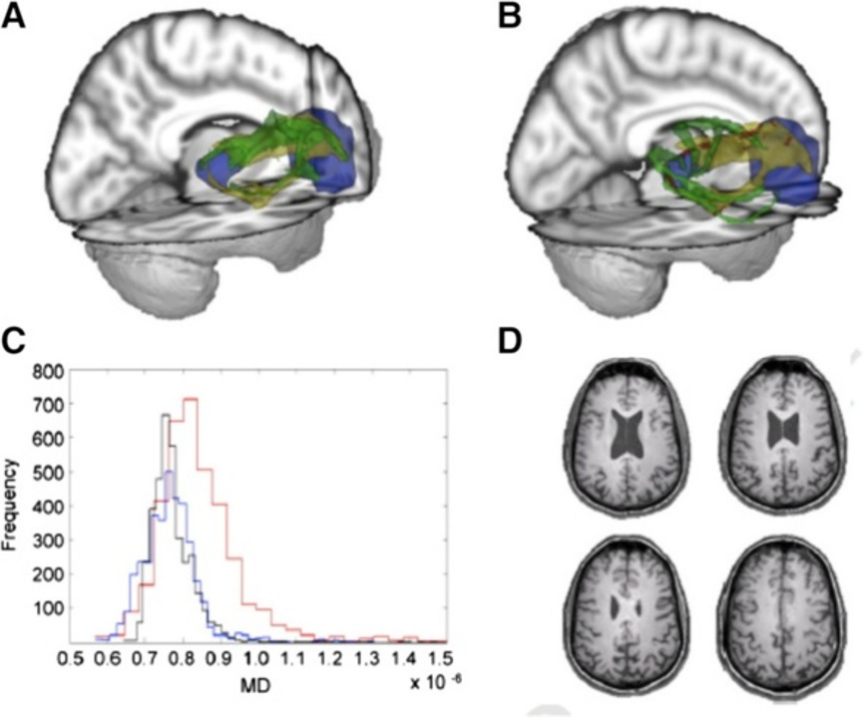
**Diffusion tensor imaging (DTI) of TBI.** Diffusion weighted volumes in 64 directions were collected from 22 patients with TBI (18 moderate/severe and 4 mild based upon Mayo classification system for TBI severity) and from 21 age-matched controls. Processing for DTI was performed and anatomic regions-of-interest were identified in controls for performing probabilistic tractography to study thalamo-cortical connections. Templates were generated that included white matter tracts isolated from probabilistic tractography. All brains were transformed into standard space and templates were applied to TBI patients and healthy controls to explore differences in white matter integrity between groups. Patients with a small **(A)** and large **(B)** number of abnormal voxels are shown. Histograms of MD values **(C)** from the patient shown in frame A (blue), frame B (red), as compared with a mean atlas **(D)** are shown. Images and data are reproduced from Squarcina *et al.*[116] with permission.

Similarly, an earlier study by Zhang and colleagues [114] using quantitative diffusion weighted imaging (DWI) found increases in the whole brain diffusion constant in professional boxers as compared to age-matched, non-boxing control subjects. The increased diffusion values were observed despite negative or nonspecific results on routine MR imaging, indicating that this imaging modality may show early pathologic changes in the cellular and microvascular structure of the brain in boxers. Zhang *et al.* [114] postulated that given the similar findings in patients with dementia, their results suggested that the whole brain diffusion coefficient represents preclinical signs of cognitive decline [114]. A later study by Zhang *et al.* [115] using DTI in a sample of 49 professional boxers and 19 healthy controls focused on the corpus callosum and the internal capsule. In that study, 42 boxers (86%) had a normal routine, clinical MRI and 7 had nonspecific white matter disease. In the boxer group, the whole brain diffusion constant was increased and FA was decreased significantly in the CC and posterior limb of IC, which the authors postulate may represent preclinical signs of subtle brain injury in professional boxers. Importantly, the increases in diffusion were found without brain abnormalities revealed on standard MR images, suggesting that diffusion tensor imaging is an important tool in the monitoring of brain function in professional boxers [113–115] and may be particularly useful in the multivariate diagnostic evaluation of individuals with CTE (Figure 10).

#### Functional magnetic resonance imaging (fMRI)

**Figure 11:**
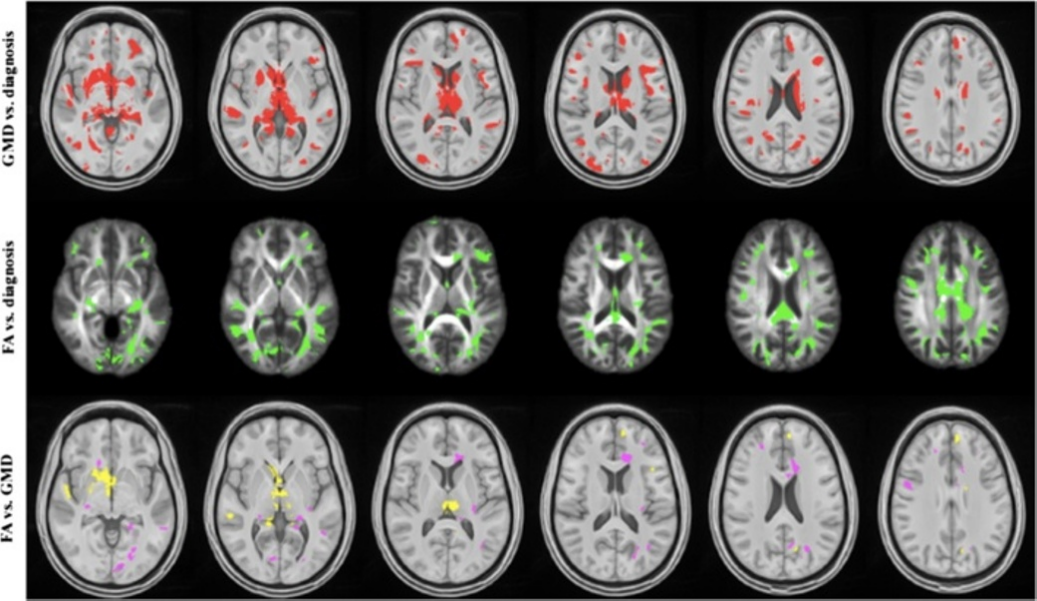
**Sparse canonical correlation analysis (SCCA) of T1-weighted MP**‐**RAGE and 30-direction diffusion tensor images (DTI) datasets are used to quantify traumatically induced disruption of WM and cortical networks.** The cohort includes 17 controls and 16 patients with TBI (age and gender matched). Each patient had a history of non‐penetrating TBI of at least moderate severity. White matter integrity is assessed by DTI and fractional anisotropy (FA) maps are generated. Separately, probabilistic segmentation of the T1‐weighted imaging is performed to assess gray matter integrity. Variation in brain shape across subjects is normalized by diffeomorphically mapping these data into a population-­specific template space. Image processing steps rely on the Camino and ANTs (Advanced Normalization Tools) neuroimage analysis open source toolkits. SCCA demonstrates significant differences between the control and patient groups in both the FA (p < 0.002) and gray matter (p < 0.01) that are widespread but largely focus on thalamocortical networks related to the limbic system. Using SCCA­identified regions, a strong correlation is identified between degree of injury in WM and GM within the patient group. Figure courtesy of James R. Stone.

fMRI involves acquisition of a blood oxygen level dependent (BOLD) sequence during performance of a specific task (Figure 11). The BOLD sequence is sensitive to conformational changes in hemoglobin and can demonstrate regional variations in blood oxygenation. It is believed these changes in blood oxygenation reflect variations in neuronal activity. However, this point is a subject of controversy given the potential for the neurovascular apparatus to be altered in states of disease.

Distinct regional differences in BOLD signal have been reported in multiple studies of concussed athletes vs. controls. Chen *et al.* [117] utilized fMRI with a working memory task to study 16 athletes (13 hockey players, 2 wrestlers, one snowboarder), most of whom had a history of > 5 career concussions. An interval of between 1 and 14 months had passed between the scan and time of last concussion. Although no differences in task performance were seen between groups, significantly different regional patterns of activation were seen within the mid-dorsolateral prefrontal cortex (MDPC) in the concussed athletes as compared to controls. In a separate study, Chen *et al.* [118] utilized an fMRI working memory task to demonstrate abnormal activation patterns in the MDPC in 9 concussed athletes as compared to controls. Of note, no significant differences were seen with routine structural imaging or behavioral outcomes between groups. Lovell *et al.* [119] employed an N-back working memory task to evaluate 28 athletes within a mean of 6.6 days after sustaining a concussion. A components analysis identified three distinct networks that were activated in both concussed athletes and controls during participation in the N-back task. One of these networks involving Brodman’s area 6 demonstrated increased levels of activity in concussed athletes compared with controls. Of note, the degree of increased activation in Brodmann’s area 6 in the concussed athletes correlated significantly with prolonged recovery time and delayed return to play.

#### Positron emission tomography (PET)

PET imaging allows for characterization of the uptake and spatial localization of radiotracers injected intravenously (IV). This imaging modality allows for the sensitive detection of small molar concentrations of radiotracers given its sensitivity for detecting the high-energy photons (512 keV) produced by radioactive decay through pair production. PET imaging in sports related TBI is currently limited and has generally been confined to use of ^18^fluoro-deoxyglucose (^18^FDG), a glucose analog that reflects metabolism. In brain, where glucose is the sole energy source, ^18^FDG is an excellent tracer for this purpose. Provenzano *et al.* [120] employed ^18^FDG PET to study 19 boxers, aged 20 to 38 yrs, and compared them to 7 age-equivalent controls. The boxers had participated in an average of 17.3 matches, had performed poorly in a match or had been knocked out, and were referred for assessment because of clinical signs of neurological impairment. The PET scans were acquired as an element of the clinical work-up. Retrospective evaluation of these scans was performed. Specific regions of hypometabolism were seen in the boxers’ brains as compared to those of the controls, including the posterior cingulate cortex, parieto-occipital cortex, frontal lobes, and cerebellum.

In addition to assessing regional metabolism, PET can be used to detect the presence of specific molecules or abnormal cellular processes (molecular imaging). Aβ·deposition is an inconsistent feature in CTE. Diffuse plaques are found in about half (47%) of neuropathologically verified CTE cases [21], a figure that represents a greater frequency than that observed acutely after severe TBI (~30%) [51]. PET imaging using Aβ probes are being currently used in large scale studies of AD. The use of amyloid imaging in suspected CTE has not been established but will be of interest given the similarity between AD and CTE phenotypes and may be useful in determining whether the presence of amyloid post TBI can help predict outcome. This will be of particular interest if the presence of amyloid is associated with worse outcomes, since interventions that reduce the trajectory of brain amyloid accumulation have already been identified in studies of AD.

The original Corsellis series identified diffuse amyloid in 50% of the cases of DP. As mentioned above, DeKosky reported acute amyloidosis within hours or days even in young patients [51]. The natural history of these deposits remains to be established, and this is an important question that can now be addressed with amyloid imaging. Menon has recently reported just such a study, and his conclusion is that the initial burst of amyloidosis is rapidly cleared [121]. One wonders, however, whether clearance might become less efficient as repeated TBI occurs, and longer term studies of such a possibility are underway in our center. In another study, about half of subjects in the chronic phase of recovery from a severe TBI showed positive amyloid scans [122].

**Figure 12:**
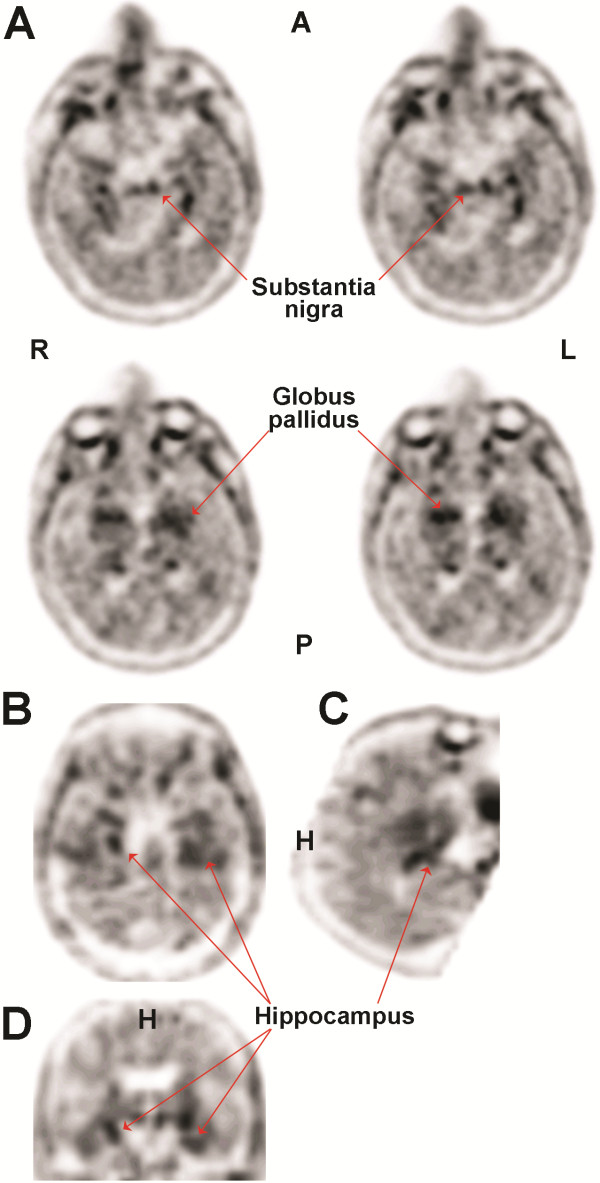
**[18F]-T807 PET imaging from a 71-year-old retired NFL player.** [18F]-T807 signals (arrows) originate from the globus pallidus (GP), substantia nigra (SN), and hippocampus. Images depict axial **(A)** sagittal **(B)** and coronal **(C and D)** orientation of the brain. A, anterior; P, posterior; L, left; R, right; H, head. Images and data are reproduced from Mitsis *et al.*[123] with permission.

Of particular relevance to CTE, PET imaging ligands are now in development for detection of abnormally phosphorylated tau associated with neurofibrillary tangles (Figure 5). Zhang *et al.* [19] recently reported development of an ^18^ F labeled compound ([^18^ F] T807) which demonstrated high affinity and selectivity for tau in competitive assays designed to assess tau binding in post-mortem human brain slices derived from individuals with AD. This study also reported high uptake and washout in rodent brains, suggesting adequate blood brain barrier penetration. Given the presence of abnormally phosphorylated tau tangles in CTE, there is great interest in exploring the utility of this probe as a potential *in vivo* marker for CTE. Successful developmdent of a molecular imaging ligand for neurofibrillary tangles would open the door for epidemiological studies to assess to the true prevalence of this disease, correlative studies to determine other potential imaging diagnostics for CTE, and as a potential metric to determine the efficacy of therapeutic or mitigation strategies for the treatment or prevention of CTE. Recent reports indicate that [^18^F] T807 has been successfully employed for the detection of CTE tauopathy *in vitro* and *in vivo* [123–125] (Figure 12).

In summary, neuroimaging in CTE holds promise in serving as an *in vivo* biomarker and should be used in adjunct with a comprehensive history and thorough clinical diagnostic evaluation in life. Prospective, longitudinal neuroimaging studies are needed to help define the clinical and biological markers associated with, and specific to, CTE. Body fluid markers may eventually complement neuroimaging markers, especially in medical centers without access to PET. These body fluid markers have recently been reviewed in detail elsewhere [126].

## Conclusions

The association of CTE with certain sports and with battlefield blast exposure provides a mandate for the construction of accurate databases and epidemiological studies. Securing valid incidence, prevalence, and relative risk data are essential. Current academic papers continue to meet with criticism that the risk is being overstated [127, 128]. While awaiting accurate data upon which to estimate relative risk, informed consent should be developed and offered to those contemplating exposure to repetitive TBI. While the administrative organizations of high impact sports and the military are the obvious organizations to begin informing potential athletes and recruits about the possible risk of CTE, another significant challenge will be the development of informed consent procedures for those overseeing the exposure of children and adolescents [27]. Finding the right balance of established fact and informed opinion will be a challenge, so as not to create unnecessary concern. Extension of genetic epidemiology studies to the pediatric and adolescent population will also be a challenge, albeit a worthy one [27]. The acquisition of definitive data about what genetic factors identified in adolescents are predictive of the eventual personal risk of CTE will require decades of study but are essential.

In parallel, application of validated laboratory models and execution of human clinical trials in the TBI/CTE area should be accelerated. Adaptation of repetitive TBI systems to the mouse holds promise for elucidating the molecular pathogenesis of CTE. The role of APOE genotype should be given high priority in both the animal and the human clinical studies [25, 27]. Compounds emerging from drug discovery efforts in academic and pharmaceutical neurodegenerative disease programs should be considered for assessment in TBI [100]. Emerging data from the AD literature indicating the key role of immune- inflammatory processes may illuminate CTE as well. New data implicate IL-1β in facilitating the development of tauopathy while reducing amyloidosis [68]. Tau oligomerization [129] may also contribute to the pathogenesis of CTE and may emerge acutely or after a delay [90]. Taken together with the Aβ independence of progressive tauopathy as reported by Brody [96], the hypothesis that tauopathy and amyloidosis can be dissociated in TBI is supported. The strong association of PTSD with TBI should also be evaluated and clarified [79].

The challenge of accelerating molecular neuropathology research in an era of dwindling resources is a major concern. Recent alliances between the NFL and the NIH, between the NFL and General Electric, and between the Department of Defense and the NIH Alzheimer’s Disease Neuroimaging Initiative provide some optimism that all the stakeholders are becoming engaged. Permanent commitment and substantial resources from these stakeholders will be required to offset the general reductions in federal support (including NIH and the Department of Defense) due to policies resulting in the recent sequestration of federal funds. In the absence of reliable prevalence data, the calculation of the potential healthcare savings by reduction of TBI exposure is not possible. The total cost of TBI exposure, of course, must take into account what may be enormous payouts from potential class action litigation verdicts. In addition to significant fiscal savings, the support of translational and clinical research programs in TBI/CTE and of development of policies that mitigate some or all of the attributable dementia risk represented by TBI/CTE is obviously justifiable on compassionate grounds. TBI, whether deliberate or unavoidable exposures, represents a source of pain and suffering that could be eliminated or at least greatly reduced with the pooled commitment and resources of the medical, sports, and military sectors. The high visibility of CTE in the media in the early 21st century makes this an opportune moment to apply the information reviewed herein so as to make important advances in elucidation of pathogenesis and in drug discovery.
